# α– Linolenic acid modulates phagocytosis and endosomal pathways of extracellular Tau in microglia

**DOI:** 10.1080/19336918.2021.1898727

**Published:** 2021-03-16

**Authors:** Smita Eknath Desale, Subashchandrabose Chinnathambi

**Affiliations:** a Neurobiology Group, Division of Biochemical Sciences, CSIR-National Chemical LaboratoryPune, India; b Academy of Scientific and Innovative Research (Acsir), Ghaziabad, India

**Keywords:** *Fatty acids*, *α- linolenic acid*, *phagocytosis*, *endosomal markers*, *MTOC repolarization*, *tauopathy*

## Abstract

Microglia, the resident immune cells, were found to be activated to inflammatory phenotype in Alzheimer’s disease (AD). The extracellular burden of amyloid-β plaques and Tau seed fabricate the activation of microglia. The seeding effect of extracellular Tau species is an emerging aspect to study about Tauopathies in AD. Tau seeds enhance the propagation of disease along with its contribution to microglia-mediated inflammation. The excessive neuroinflammation cumulatively hampers phagocytic function of microglia reducing the clearance of extracellular protein aggregates. Omega-3 fatty acids, especially docosahexaenoic acid and eicosapentaenoic acid, are recognized to induce anti-inflammatory phenotype of microglia. In addition to increased cytokine production, omega-3 fatty acids enhance phagocytic receptors expression in microglia. In this study, we have observed the phagocytosis of extracellular Tau in the presence of α-linolenic acid (ALA). The increased phagocytosis of extracellular Tau monomer and aggregates have been observed upon ALA exposure to microglia cells. After internalization, the degradation status of Tau has been studied with early and late endosomal markers Rab5 and Rab7. Further, the lysosome-mediated degradation of internalized Tau was studied with LAMP-2A, a lysosome marker. The enhanced migratory ability in the presence of ALA could be beneficial for microglia to access the target and clear it. The increased migration of microglia was found to induce the microtubule-organizing center repolarization. The data indicate that the dietary fatty acids ALA could significantly enhance phagocytosis and intracellular degradation of internalized Tau. Our results suggest that microglia could be influenced to reduce extracellular Tau seed with dietary fatty acids.

## Introduction

In the central nervous system (CNS), embryonic mesoderm-derived microglia is the major group of resident immune cells, which consists of 20% of the total glial population [1]. In a physiological condition, microglia displays ramified morphology having long branched cellular processes, which senses the tissue damage, pathogenic invasions, etc. [2,3]. The surveillant stage of microglia is maintained by neuronal and astrocytes-derived factors [4–7]. On external stimuli, microglia get activated, and the ramified morphology changes to amoeboid morphology. Microglia are either classically activated to give a pro-inflammatory response or follow alternative activation to show anti-inflammatory response. Alzheimer’s disease (AD), which is a progressive neurodegenerative disease, shows a predominance of inflammatory microglia [8,9]. Gliosis in AD pathology indicates abnormal morphology, excessive activation of microglia, and astrocytes [10]. Neuroinflammation acts as a key triggering process in AD, where amyloid-β and neurofibrillary tangles of Tau are found to be surrounded by microglia [11,12]. The accumulation of aggregated proteins and the presence of inflammatory cytokines such as IL-1β, TNF-α, and IFN-γ promote proinflammatory phenotype of microglia, which alters phagocytic nature of microglia. The phagocytic state of microglia is regulated by the expression of receptors on the cell surface, membrane fluidity, downstream signaling, and rearrangement of actin network [7,13]. Phagocytic ability of microglia is under the influence of various environmental factors such as lipids, lipopolysaccharides (LPS), and cytokines [14,15]. Dietary lipids affect the brain extensively since fatty acids are the building blocks of the brain. Dietary fatty acids, primarily polyunsaturated fatty acids including omega-3 fatty acids docosahexaenoic acid (DHA-22 3 n:6), eicosapentaenoic acid (EPA- 20 3 n:5), and α-linolenic acid (ALA 18 3 n:3), have beneficial effects on the brain [14]. Omega-3 fatty acid enhances the fluidity of cell membrane by incorporating long-chain fatty acids into phospholipids of cell membrane. The increased fluidity of the cell membrane holds the extent of receptor expression on the cell surface and their downstream signaling [16–19]. DHA and EPA are either taken up by dietary lipids or synthesized by ALA in the body. DHA and EPA are the main regulators of lipid mediators that drive the resolution phase by suppressing the inflammatory response and helps to restore the homeostasis [18,20,21].

The establishment of Tau as a factor of neurotoxicity and neuroinflammation is still a matter of debate, but recently accepted concept of Tau as a prion-like protein supports this hypothesis [22–24]. The spreading of Tau and its ability to cause template-dependent deformation in the healthy neuron can be targeted [25–27]. Omega-3 fatty acids are found to implement the suppression of neuroinflammation and trigger polarization of microglia [28–30]. Omega-3 fatty acid elevates the resolution phase and mediates tissue repair, healing, clearing of debris and maintains homeostasis by microglia [22,29]. Enhanced phagocytic nature of microglial cells due to the exposure of omega-3 fatty acids could act as a therapeutic strategy to minimize the spreading of Tau [25,31]. In the phagocytosis or degradation process, Rab proteins, especially Rab5 and Rab7, play an important role in the intracellular vesicle trafficking and mediate the endocytic pathways. The defective degradation of proteins further attenuates phagocytosis, resulting in excessive intracellular load [32]. Upon uptake the phagosome merges with the sorting endosome to undergo degradation process via endosomal acidification [33,34]. Rab5 is associated with the early endosomes, whereas Rab7 identifies late endosomes in the phagocytosis process [35–38]. Hence, to study the internalization and the subsequent degradation of Tau, Rab5, Rab7, and their transition would help to provide the insights of the process [39,40]. The final step of phagocytosis involves a fusion of late endosomes with lysosome to form a phagolysosome as a microcidal compartment. The fusion of late-endosome involves lysosome-associated membrane proteins (LAMP) and other luminal proteases [32,41–44].

Microglia activation leads to polarization and migrates in a particular direction, depending upon the directional clues. Cytokines and chemokines response to play an important role in migration and polarization of microglia through CX3CL1-CX3CR1, IL-4, CCR5, CCR3, and CCR1-mediated signaling [7,45,46]. The polarized state of microglia is maintained by the cytoskeletal network wherein actin provides directional sensing and microtubule dynamics for the mechanical strength to move cell forward [47]. In this study, we observed the enhanced phagocytosis in the presence of ALA and subsequent degradation of internalized Tau, which was confirmed by Rab5, Rab7, and LAMP2A. On the other hand, ALA can improve migration profile of microglia that might help the phagocytosis process.

## Results

### Tau aggregation in the presence of ALA

**Figure 1.**
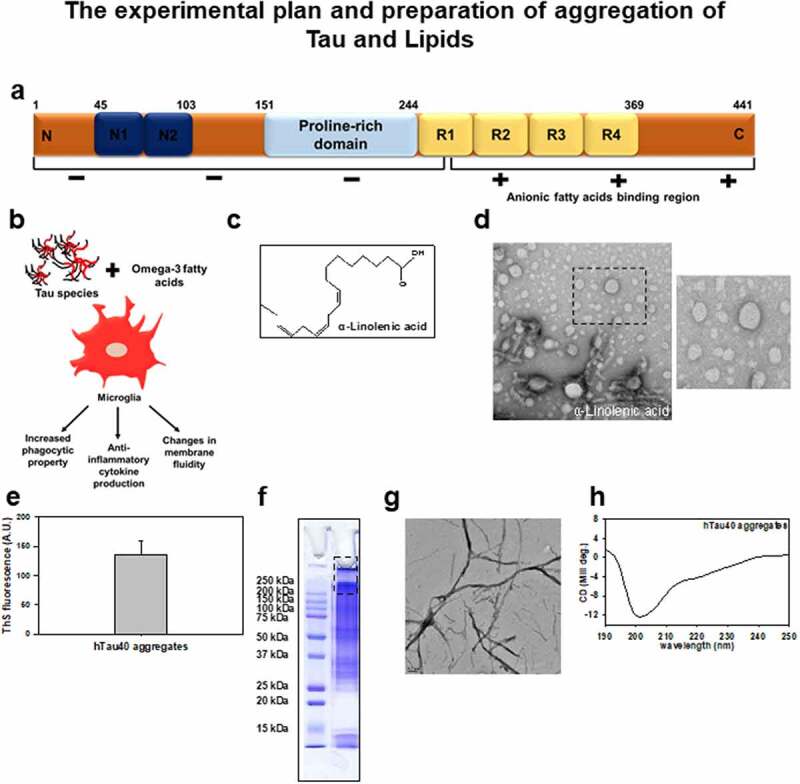
**Biochemical characterization of hTau40 aggregates**. Experimental approach and biochemical characterization of ALA: (a) Tau structure bar diagram showing domains of hTau40 having 441 amino acid sequence and specified with the distribution of net charge domain vise. The fatty acid binding region is indicated at repeat region of Tau structure. (b) The proposed hypothesis for the effect of ALA and hTau40 species on microglia, ALA changes the membrane composition of microglia and enhances anti-inflammatory phenotype with increased phagocytic capacity, and also modulates membrane fluidity; we propose that increased phagocytic ability would clear the extracellular Tau species. (c) Chain structure of α-linolenic acid (ALA) (18 3 n: 3). (d) ALA was dissolved in 100% ethanol and solubilized at 50°C for 2 h. The microscopic observation of ALA vesicles was done by transmission electron microscopy for the morphological analysis. The enlarged area showing zoomed images of vesicles; scale bar is 200 nm. (e) ThS fluorescence assay, to observe the aggregation propensity of hTau40 at 120 h time points in the presence of heparin *in vitro*. (f) SDS PAGE analysis of hTau40 aggregates. The marking of 250 kDa shows higher order bands corresponding to aggregates. (g) TEM analysis of hTau40 aggregates after 120 h, scale bar is 0.2 µm. (h) CD analysis to study the conformation changes of Tau on aggregation from random coiled to β-sheet structure. The spectra were analyzed between 250 and 190 nm range

ALA is an essential omega-3 fatty acid, which is a precursor of DHA and EPA [48]. In this study, we aim to understand the role of ALA on the function of microglia and its effect on extracellular Tau in AD. Tau, a natively unfolded protein, stabilizes microtubules in neuron and other CNS cells. The longest isoform of Tau has 441 amino acids with two inserts, proline-rich domain, and four imperfect repeat regions (Figure 1a). The positive charge of the repeat region of Tau facilitates the binding of anionic free fatty acids [49]. The beneficial effect of ALA as a potent anti-inflammatory agent and a precursor of other omega-3 fatty acids DHA, EPA provides therapeutic strategy in AD. In this study, we explored the neuroprotective anti-inflammatory role of ALA on exposure to microglia and its effect on phagocytosis of extracellular Tau (Figure 1b). ALA is a polyunsaturated omega-3 fatty acid (18:3 n-3) having three double bonds in its structure (Figure 1c). For the preparation of ALA, it was dissolved in 100% ethanol and then solubilized at 50°C, which produce irregular vesicles-like structure, which has been shown in transmission microscopy (TEM) images (scale bar is 200 nm) (Figure 1d). Due to high hydrophilic nature, high net positive charge and lack of hydrophobic residues account for the natively unfolded nature of Tau. This flexible structure of Tau due to unfolded nature aids in microtubule-binding and stability. The highly soluble form of Tau can be induced to aggregate in the presence of polyanionic agents such as heparin, which neutralize net positive charge of Tau *in vitro*. The hTau40 aggregates produced *in vitro* with heparin and their characterization with different biochemical assays are enlisted in Figure 1e–h. Free fatty acids such as arachidonic acid induce spontaneous self-assembly of Tau protein to form aggregates in a dose-dependent manner [49]. *In vitro* aggregation of hTau40 in the presence of heparin was confirmed with ThS fluorescence for time period of 120 h, which ranges from 140 to 160 fluorescence units. SDS PAGE analysis was performed, which showed higher order Tau aggregates bands at 200 kDa and above and TEM (scale bar is 0.2 μm) for visualization of aggregated Tau fibrils (Figure 1e–g). The confirmation for the aggregates formation in the presence of Tau was carried out with the circular dichroism spectroscopy (CD). The native random coil nature of Tau changes to β-sheet conformation on formation of aggregates can be detected with the shift in absorbance above 200 nm in CD data (Figure 1h).

## Internalization of extracellular Tau in the presence of ALA in microglia

**Figure 2.**
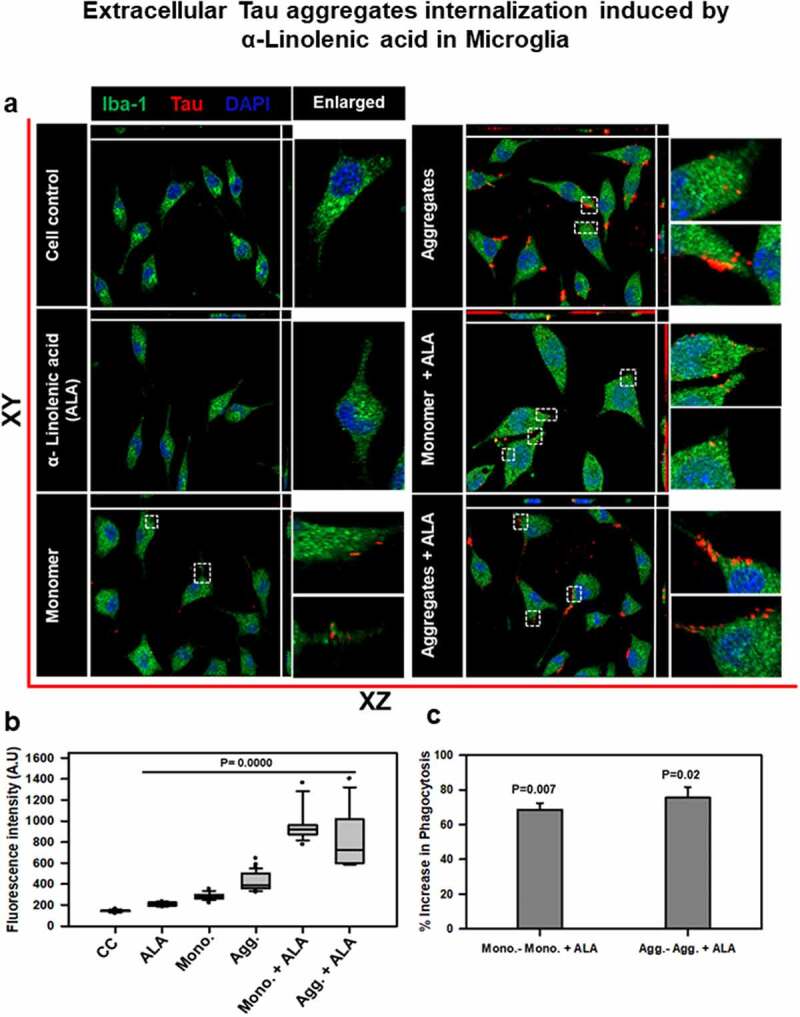
**Extracellular Tau aggregates internalization, induced by α-linolenic acid in microglia**. Internalization of hTau40 recombinant Tau in Iba-1 positive microglia. (a) Cells were incubated with hTau40 aggregates species, hTau40 monomer species (1 µM) alone, and along with the α-linolenic acid (40 µM) for 24 h at 37°C. The cells were fixed after 24 h and stained with anti-Iba-1 antibody (green) and T46 Tau antibody (red) and observed by fluorescence microscopy; scale bar is 20 µm. The orthogonal images denote the internalization of Tau, indicated at X and Y plane to visualize the intracellular position. The enlarged panel indicates internalized Tau. The images were taken with the Zeiss fluorescence microscope with Apotome 2.0. (b) Quantification of mean intensity of internalized Tau in microglia cells showing the extent of internalization Tau in microglia cells, which is highly significant (*P* < 0.001) compared with cell control (no treatment) and ALA-treated cells. (c) Percentage increase in phagocytosis of hTau40 in microglia after ALA exposure to cells; percentage increase in aggregates compared to aggregates with ALA-exposed groups and in monomer compared to monomer with ALA groups calculated from the intracellular intensity of Tau among the groups; significance is *P* = 0.02 and 0.007, respectively

We expose microglia cells (N9) with 40 µM ALA for 24 h and checked for the internalization of extracellular Tau monomer and aggregates (Figure 2a). N9 cells were treated with 40 µM ALA as a control, 1 µM Tau monomer, aggregates, and their respective treatment with ALA. Immunofluorescence staining was performed to study the internalization of Tau (red) in Iba-1 (green)-positive microglia cells since Iba1 is a marker for microglia and involves in membrane-ruffling and phagocytosis [50,51]. The orthogonal images indicate intracellular localization of Tau in X and Y plane; the enlarged area denotes respective internalized Tau (Figure 2a). The mean intensity of Tau inside the microglia was calculated by ZEN 2.3 software. The intensity of Tau has been found to be increased in monomer, aggregates, and ALA exposure groups compared to other treatment groups (Figure 2b). The fluorescence intensity of internalized Tau (monomer and aggregates) in the presence of ALA was found to elevate to 800–1400 fluorescence units compared to other control groups. Whereas microglia exposed to Tau species (monomer and aggregates) indicated the intracellular intensity of Tau between 200 and 600 fluorescence units. The internalization of extracellular aggregates was observed to be increased compared to extracellular monomer, thus indicating that ALA enhances phagocytosis ability of N9 cells. Intracellular intensity of internalized Tau was quantified; the results have significantly increased in cells treated with ALA compared to control (no treatment) cells (*P* < 0.001) (Figure 2b). ALA exposure increased the intrinsic phagocytic capacity of microglia in monomer and aggregates by 68% and 75% (*P* < 0.05, 0.01), respectively (Figure 2c). This indicates five- to six-fold increase in intrinsic phagocytic ability of microglia. Supplementary figure 1 incorporates the individual panel for all the filters given in the merge images for better understanding of morphology and immunofluorescence staining as Tau (red), Iba-1 (green), DAPI (blue), and differential interference contrast (DIC) (Fig. S1).

## Effect of ALA on endosomal trafficking of internalized Tau and its degradation pathway

**Figure 3.**
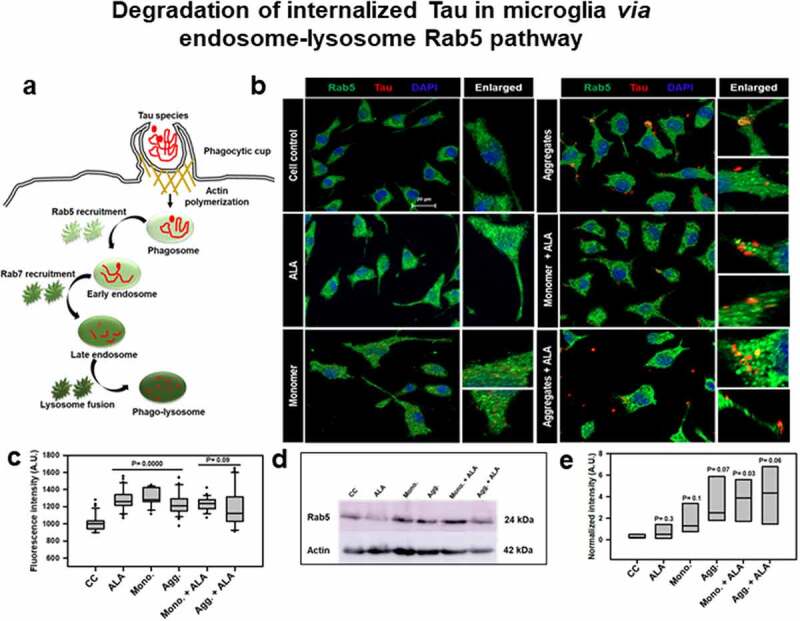
**Degradation of internalized Tau in microglia via endosome–lysosome Rab5 pathway**. A microglia cell was exposed to hTau40 monomer and aggregates in the presence and absence of ALA and observed for the levels of Rab5 (green) and Tau (red) by fluorescence microscopy. The degradation of internalized Tau was studied with the early endosomal marker and late endosomal markers. (a) In the pathway, the maturation of phagocytic vesicle takes place that can be marked with the early endosomal marker Rab5 and late endosomal marker Rab7. We observed the colocalization of internalized Tau with endosomal markers (Rab5) to trace the degradation of internalized Tau. (b) The fluorescence microscopy images indicate the levels of endosomal markers and their colocalization with internalized Tau. The enlarged panel indicates specific area in the cells showing colocalization between Rab5 and Tau; scale bar is 20 μm. (c) The quantification of mean intensity of endosomal markers was carried out with ZEN 2.3 software; significance is *P* < 0.05. (d) Expression analysis of early endosomal marker (Rab5) was observed by western blot after various treatments of hTau40 monomer, aggregates, and ALA after 24 h. (e) Quantification of protein bands is plotted as intensity and normalized with β-actin as a loading control

**Figure 4.**
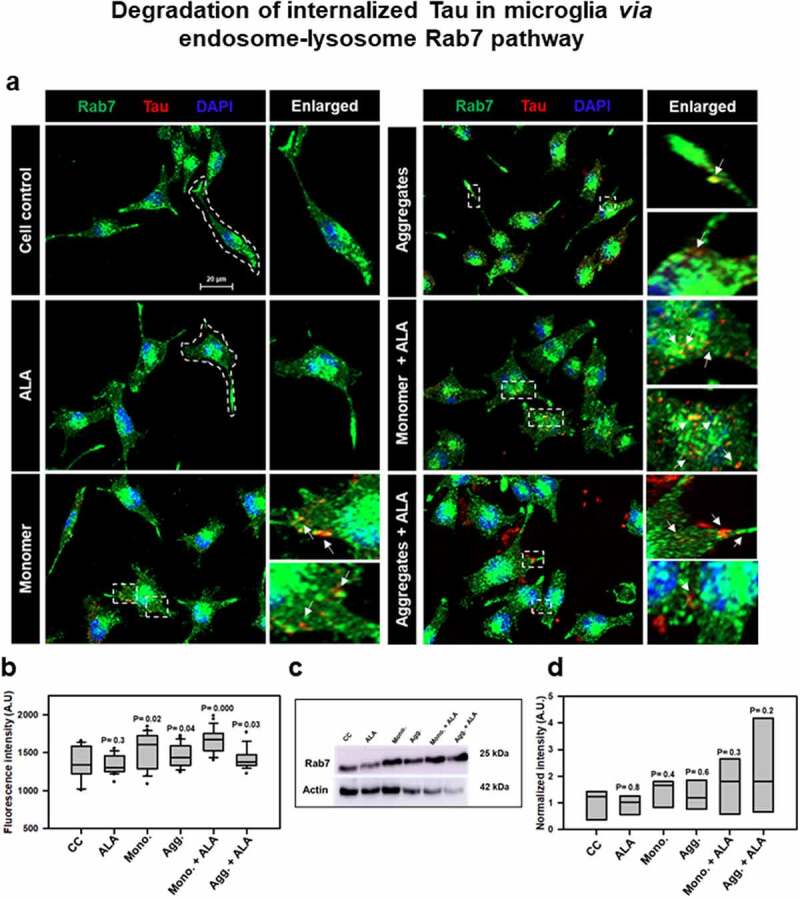
**Degradation of internalized Tau in microglia via endosome–lysosme Rab7 pathway**. Fate of internalized Tau was observed with the help of late endosomal marker Rab7 by fluorescence microscopy. (a) The colocalization of internalized Tau was observed with late endosomal marker Rab7, and the enlarged area indicates the specific area of colocalization inside the cell. The white arrowmarks denote the position and colocalization of internalized Tau with Rab7; scale bar is 20 μm. (b) The intensity analysis of Rab7 plotted as a mean intensity of Rab7; significance is *P* < 0.05. (c) Expression profile of Rab7 was analyzed by western blot after exposure of extracellular Tau. (d) The quantification of protein bands was carried out by calculating the intensity and normalized with β-actin as a loading control

The phagosomes after internalization were subjected to lysosome-mediated degradation via endosomal maturation process in phagocytosis [6,52]. The internalized phagosome further fuses with endosomal compartments, which is mediated by endosomal markers Rab5 and Rab7. The endosomal maturation step ends when the phagosome fuses with lysosome, where the enzyme-mediated degradation of microorganisms occur in immune cells. We hypothesize that the degradation of internalized Tau could be evaluated by its colocalization with endosomal compartments. We studied downstream early and late endosomal markers, Rab5, Rab7, and LAMP-2A, respectively, for the colocalization with internalized extracellular Tau (Figure 3a). The immunofluorescence images of Tau and endosomal or lysosomal markers after 24 h of exposure with extracellular Tau monomer, aggregates, and ALA were observed. The results shows the levels of endosomal and lysosomal markers in the cell and its colocalization with internalized Tau are represented with white arrow marks in the respective images (Figures 3b and 4a). The enlarged images show the particular area indicating colocalization between internalized Tau with Rab5 and Rab7 in microglia (Figures 3b and 4a). The intracellular mean intensity of Rab5 and Rab7 was estimated by ZEN 2.3 software. The expression of Rab5 was found to increase significantly in case of aggregates with ALA-treated cells that ranged from 1000 to 1600 fluorescence units (*P* < 0.001), whereas Rab7 showed increased levels of protein with ALA treatment in both monomer and aggregates-treated cells compared to other control groups (*P* < 0.001) (Figures 3c and 4b). Expression of Rab5 and Rab7 was also checked with western blot, and aggregates and ALA-treated groups were found to increase exceptionally with respect to both Rab5 and Rab7 protein expression (Figures 3d e)and 4c,d. The intracellular intensity of Rab7 quantified by ZEN 2.3 software was found to increase in Tau-exposed groups (monomer and aggregates) in the presence of ALA, which ranges from 1200 to 1800 fluorescence units (Figure 4b). The western blot quantification for both Rab5 and Rab7 indicated the increased expression of protein with respect to monomer, aggregates exposed along with ALA (Figures 3e and 4d). The increased levels of Rab5 and Rab7 in case of ALA-treated cells in both monomer and aggregates show cells are undergoing more of phagocytosis and the internalized Tau is channelizing toward degradation pathway. The mature late-endosome containing target protein then fuses with the lysosome. The high pH in lysosome compartment and other hydrolytic enzymes containing proteases, lipases, lysozymes, and cathepsins induce degradation of internalized targets [53].

## Effect of ALA on lysosome-mediated degradation of internalized Tau

**Figure 5.**
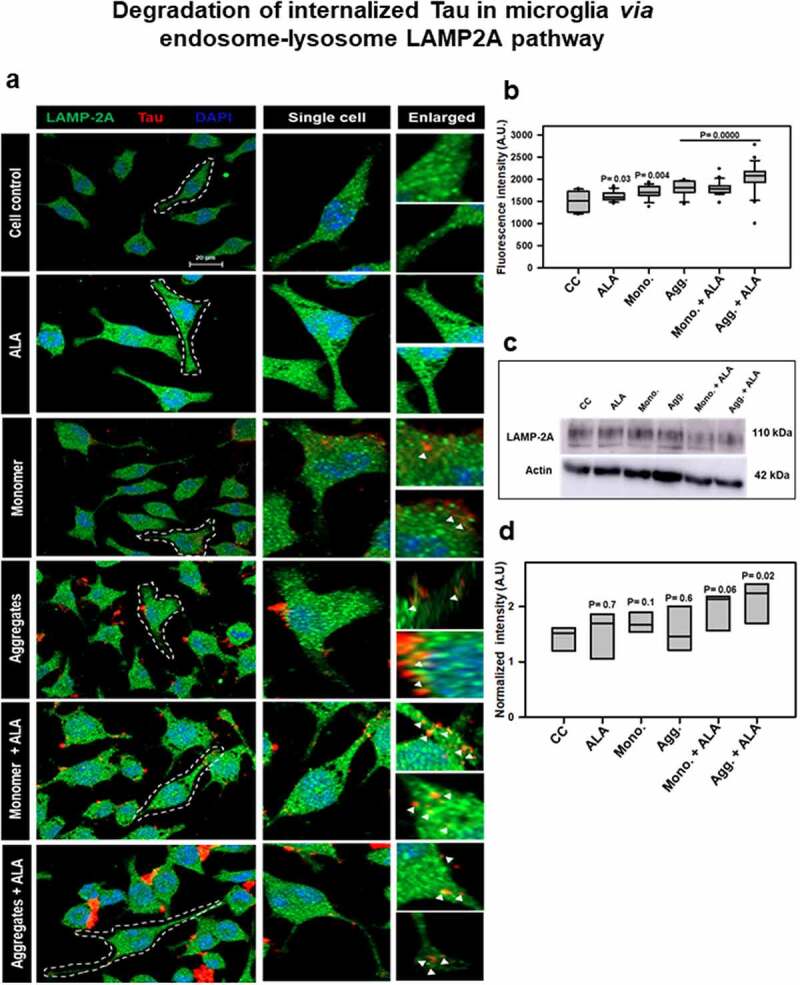
**Degradation of internalized Tau in microglia via endosome–lysosome LAMP-2A pathway**. Last step of degaradation pathway includes fusion of late endosome with lysosome studied by fluorescence microscopy. (a) Internalization of extracellular Tau (red) studied with its colocalization with LAMP-2A (green) after 24 h of ALA and extracellular Tau exposure. The images indicate levels of LAMP-2A and its colocalization with Tau. Enlarged panel indicates that specific area from the representative image showing colocalization of Tau and LAMP-2A, indicated with white triangles. Scale bar is 20 μm. (b) Intracellular intensity of LAMP-2A was calculated from immunofluorescence images and plotted as mean intensity inside the cell. The significance is *P* < 0.05. (c) Levels of LAMP-2A were detected by western blot after ALA and Tau exposure. (d) Quantification of intensity of protein bands, normalized with the β-actin as a loading control

Further to understand lysosome-mediated degradation, N9 cells were treated with extracellular Tau monomer, aggregates, and ALA, and the cells were stained for immunofluorescence analysis with Tau (red) and LAMP-2A (green) post treatment. The levels of LAMP-2A and its colocalization with internalized Tau are indicated through immunofluorescence analysis (Figure 5a). The representation of immunofluorescence images indicated colocalization of Tau and LAMP-2A; enlarged image panel indicates colocalization spotted with white arrow marks (Figure 5a). The intracellular intensity of LAMP-2A was calculated, which showed extensive increase of LAMP2A mean intensity in aggregates with ALA group compared to other treatment groups that corresponds to 1500–2300 fluorescence units (Figure 5b). The levels of LAMP-2A by western blot were analyzed, and the ALA-treated N9 cells showed an increase in levels in both Tau monomer and aggregates-exposed cells (Figure 5c, d).

## ALA enhances migration of microglia

**Figure 6.**
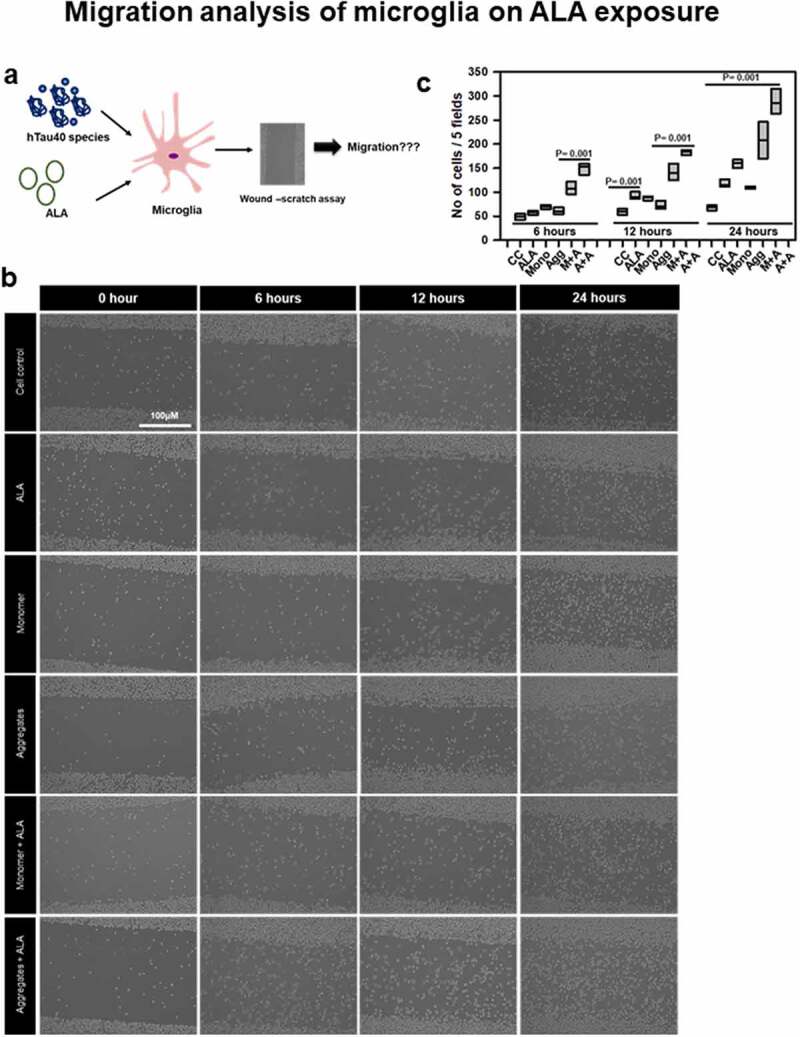
**Migration analysis of microglia in the presence of ALA**. Increased migration microglia is a key property of anti-inflammatory phenotype. Observe the effect of ALA on migration of microglia since omega-3 fatty acids enhance anti-inflammatory phenotype. (a) ALA was observed to enhance the phagocytosis of microglia that is also assisted by migration of microglia. The effect of ALA on migration under the influence of hTau40 monomer and aggregates has been studied. (b) The migration of microglia was studied by wound-scratch assay. Migration of cells into the scratch was studied with different time intervals 0, 6, 12, and 24 h after the scratch, which is observed with phase contrast microscope. Scale bar is 100 μm. (c) In each treatment groups, randomly five fields were chosen and the number of cells migrated into wound was counted. The comparison for each time point was carried out with its respective time point control (no treatment) group. The data are plotted as migration of each group at particular time point; significance is *P* < 0.001

Omega-3 fatty acid induces alternative anti-inflammatory phenotype of microglia; that depicts increased migration of microglia. The alternative activation observed with IL-4 treatment to microglia induces excessive migration [54]. We hypothesize that exposure of ALA will modulate the cell membrane and increased its fluidity, which shows the increased migration of N9 cells (Figure 6a). In this study, we checked for migration ability of microglia by wound-scratch assay. Time-dependent migration of microglia was studied in the presence of ALA for 0, 6, 12, and 24 h (Figure 6b). We quantified the number of cells into the wound after every time point by phase contrast microscopy. ALA was found to increase the migration of microglia compared to cell control. At 24 h, time point monomer showed higher migration rate (120–150 cells/field) than aggregates (80–100 cells/field); however, their respective exposure with ALA enhanced the migration to a greater extent. The migration profile at 24 h was found to be highest in aggregates with ALA condition (250–300 cells/field) (Figure 6c). Exposure of ALA alone in microglia was found to increase migration of cells compared to cell control (100–120 cells/field). These results suggest that the ALA supports microglia to increase the migration, which is one of the key properties of anti-inflammatory phenotype of microglia.

## ALA polarizes nuclear-centrosomal axis in microglia

**Figure 7.**
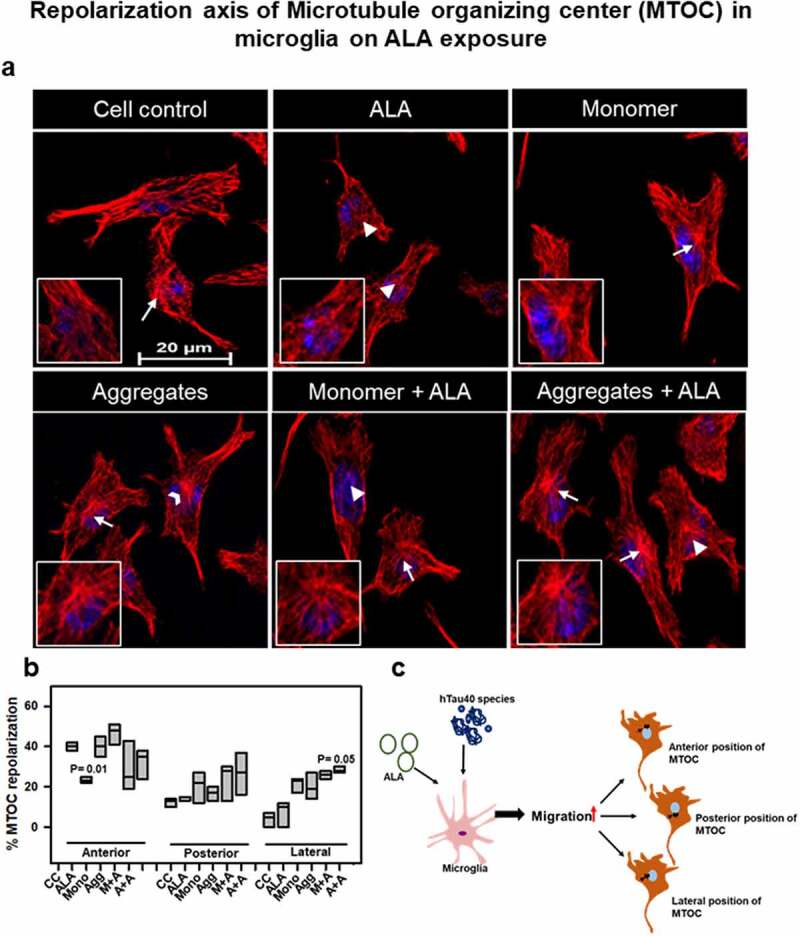
**Repolarization of axis of MTOC on ALA exposure**. (a) Microglia were treated with hTau40 monomer, aggregates, and ALA for 24 h and observed for MTOC positioning with respect to nucleus in N9 cells. Fluorescence microscopy images were analyzed for MTOC positions stained with α-tubulin (red) and DAPI. The panels showing merge images with different MTOC position white triangle (anterior position), white open arrowhead (posterior position), and white arrow (lateral position). The inlet indicates the enlarged area showing MTOC in the cell. Scale bar is 20 µm. (b) Quantification of MTOC reorientation. The percentage number of cells for the different positions of MTOC was calculated in 10 different fields in all the treated groups. (c) Exposure of ALA with hTau40 monomer and aggregates was found to increase the migration that modulates the orientation of MTOC positions in the cells. The expected different positions of MTOC are depicted according to pectoral representation

Increased migration is also associated with the repolarization of MTOC along the nucleus centrosome (NC) axis in the cell. In migratory cells, the microtubule network reorients to migratory leading end for the forward motion, and hence the MTOC positions are found predominantly in the anterior region of nucleus. In case of highly migratory cells, the repolarization of MTOC is observed to be present on different positions such as anterior, posterior, and lateral positions of nucleus. The microtubule network was found to be dense at the nucleus, spreaded toward lamellum, and bundled down to uropod at rear end (Figure 7a). Microglia cells irrespective of any treatment condition showed maximum percentage of cells showing anterior position of MTOC (35–60%) with respect to nucleus. In case of ALA-treated cells in both monomers and aggregates showed MTOC orientation in all the different positions, whereas in other treatment groups anterior position of MTOC prevails (Figure 7a). The posterior and lateral positions of MTOC (20–35% each) with respect to the nucleus were found to increase in Tau-exposed (monomer and aggregates) groups in the presence of ALA. In case of aggregates with ALA treatment, the percentage occurrence of different positions of MTOC around the nucleus is near to equal (Figure 7b). The data also suggest that, upon ALA exposure, the lateral position of MTOC around the nucleus has increased; however, in other treatment groups the anterior position is being favored by cells (Figure 7b). The picture representation suggests that ALA treatment to microglia reorients the MTOC to different positions anterior, posterior, and lateral to nucleus unlike unipolar control cells (Figure 7c). All the panels of immunofluorescence images with DIC are shown, indicating the exact positions of MTOC (Fig. S2).

## Discussion

**Figure 8.**
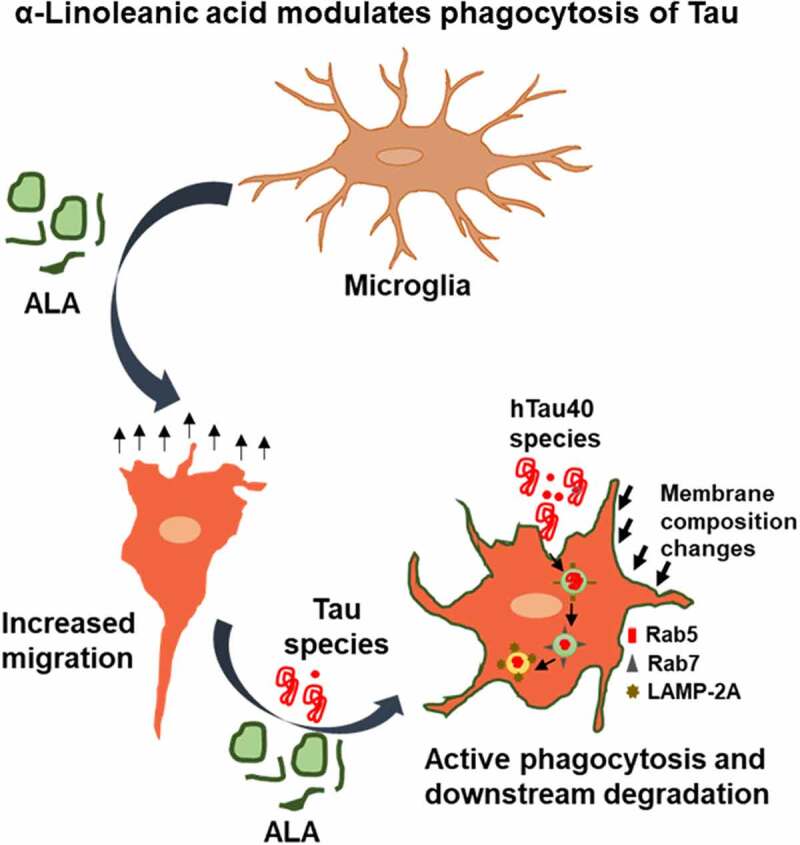
**ALA modulates phagocytosis of Tau**. ALA after exposure to N9 cells enhances extracellular Tau phagocytosis and its lysosome-mediated degradation. Thus it reduces accumulation internalized Tau in microglia. On the other hand, ALA enhances migration property of microglia to locate the target and undergo phagocytosis of extracellular Tau

The extracellular Tau species after recognition by immune cells induces the immune response [55]. Omega-3 fatty acids have the ability to effectively dampen the response given by immune cells [56]. Dietary omega-3 fatty acids induce microglial anti-inflammatory immune response, which would enhance the clearance of extracellular pathological Tau species [22]. In the present study, N9 microglia cells were exposed to ALA, being an omega-3 fatty acid and observed for its beneficial effects [57]. Exposure of ALA enhanced phagocytic ability of microglia by internalizing extracellular Tau species. The effect of ALA on migration has been studied in microglia cells as they assist the phagocytosis process. The enhanced phagocytosis in the presence of ALA should also channel the internalized antigen toward lysosome-mediated degradation for the desired clearance of extracellular antigens. The degradation of internalized Tau was denoted with the endosomal markers and their colocalization with internalized Tau. The reported results suggest the beneficial effects of ALA in the brain (Figure 8).

In previous studies, it has been proven that the seeding nature of Tau causes template-dependent aggregation on uptake by healthy neurons [25]. The aggregated extracellular Tau species secreted by various mechanisms have the tendency to propagate the disease [27,58,59]. The use of other omega-3 fatty acids, DHA and EPA, has been studied for the uptake of extracellular Aβ-plaques and their clearance [31,60]. The insoluble pathological aggregated form of Tau prepared *in vitro* to study the phagocytosis was confirmed by SDS PAGE, TEM, ThS fluorescence, and CD analysis. To understand the effect given by ALA, optimum concentration of ALA was used for the cellular studies [31,49]. To evaluate the beneficial role of ALA for the uptake of extracellular Tau, we had incubated Tau with N9 microglia cells for 24 h. The increased phagocytosis of extracellular Tau has been observed with ALA treatment conditions [61]. N9 cells treated with Tau monomer and aggregates along with ALA were found to increase the phagocytosis by more than 60% of total activity. This result indicates the improved ability of microglia to clear extracellular Tau. Omega-3 fatty acids exert anti-inflammatory properties to cells due to their ability to produce specialized pro-resolving molecules (SPM), which on attending certain concentration shows their effects [62]. DHA and EPA are the main omega-3 fatty acids that increase the microglial activation and act as a main precursor of SPM; they are also found to activate PPAR-γ to mediate anti-inflammatory response [28,63]. The clearance of extracellular targeting species in case of AD could be objectified with dietary omega-3 fatty acids. AD is being characterized by the endo-lysosomal abnormalities and accumulation of Rab5-positive enlarged endosomes followed by detectable Aβ-plaques [40,64]. The accumulation impairs the fusion of autophagosomes with late-endosomes and lysosomal degradation. The transition of phagosomes from Rab5 to Rab7 is one of the important events, which specifies the degradation of internalized antigens [65]. In our results, the levels of Rab5 and Rab7 were found to increase in case of ALA treatment, and a significant colocalization of internalized Tau with Rab5 and Rab7 was observed. The results indicate that the internalized Tau is undergoing degradation pathway instead of accumulating inside the cell. Increased expression of Rab5 and Rab7 observed via both fluorescence images and western blot experiments with respect to Tau species and ALA group confirms the fact that the internalized Tau is channelizing toward degradation pathway. The final step of phagosome maturation ends up with the fusion with lysosome. The formation of phagolysosome is regulated by lysosomal-associated membrane proteins. Double knockout of LAMP-2A was found to impair the maturation of phagosome by halting the process prior to acquisition of Rab7 and affects lysosome density in cell [53,66]. LAMP-2A might be a better target to study the lysosomal degradation of internalized Tau. We have observed from the results that ALA enhances LAMP-2A levels in cell, and its colocalization with internalized Tau indicates the active phagocytosis. Elevated levels of LAMP-2A suggest active expression of lysosome and its colocalization with Tau supplement the information that ALA might induce the phagolysosome formation for clearance of internalized Tau.

Activation stage of microglia upon stimulation is observed with increased migration, phagocytosis, proliferation, and cell shape changes, which are assisted by actin cytoskeleton [67,68]. The cell migration profile for N9 cells treated with different groups was studied by wound-scratch assay. Excessive migration was seen with the ALA treatment compared to control groups. The increased migration of microglia, especially with Tau species and ALA,indicates the excessive activation of cells, which might be useful for the phagocytosis process to engulf the extracellular targets. The protrusive and contractile force needed for the migration is supported by actin rearrangements. Whereas polarization of microglia is supported by both actin and microtubule cytoskeleton. In migratory polarized microglia, well-assisted reorientation of NC axis is observed. Migratory cells show anterior NC axis where MTOC, endoplasmic reticulum, and Golgi apparatus are in front of nucleus, which stabilized the front end [69]. The ‘posterior’ position of MTOC is rare, but observed in some migratory immune cells such as neutrophils [70]. The specific regulation of MTOC positions is unknown, but it can be regulated by extracellular signals such as chemotaxis [71]. However, in highly migratory ALA-treated cells it lacks the preference of NC axis and other positions such as posterior and lateral were favored [54,69]. This is also observed in highly migratory immune cells such as neutrophils and T-lymphocytes. The increased migration supports enhanced phagocytosis in microglia [70].

## Conclusions

The induced phagocytosis of extracellular Tau followed by its degradation via endo-lysosomal pathway shows the beneficial and neuroprotective role of ALA. This suggests the potential role of ALA to impose anti-inflammatory property of microglia in a disease condition. The phagocytosis is coupled with the degradation of internalized Tau via lysosome-mediated degradation. The phagocytosis and degradation pathway is also supplemented with enhanced migration, which extends the neuroprotective function of microglia. This indicates the potentially beneficial role of dietary supplement of ALA over Tau seeding (Figure 8).

### Materials and methods

#### Chemicals and primary antibodies

*Solvents*: Ethanol (cat. no. 180077), isopropanol (cat. no. 194006), and methanol (cat. no. 201002) were purchased from MP Biomedicals. *Protein purification reagents*: NaCl, phenylmethylsulfonylfluoride, MgCl_2_, APS, and DMSO are from MP Biomedicals. SDS is from Sigma. Tris base, 40% acrylamide, and TEMED are from Invitrogen. MES and BES are from Invitrogen. IPTG and dithiothreitol (DTT) are from Calbiochem. Protease inhibitor cocktail and EGTA are from Invitrogen. Luria–Bertani broth (Himedia) and ampicillin are from MP Biomedicals. *Cell culture*: Phosphate buffer saline (PBS) and radioimmunoprecipitation (RIPA) buffer are from Invitrogen. ALA (L2376) is from Sigma. The N9 microglial cell line no. is CVCL- 0452, Roswell Park Memorial Institute (RPMI), fetal bovine serum (FBS), horse serum, trypsin-EDTA, and penicillinstreptomycin were also purchased from Invitrogen. MTT reagent and Triton X-100, Trypan-Blue were purchased from Sigma. The coverslip of 12 mm was purchased from Bluestar for immunofluorescence. Copper-coated carbon grids for TEM analysis were purchased from Ted Pella, Inc. *Antibodies*: - In immunofluorescence and western blot study, we used the following antibodies: βb-actin (Thermo Fisher cat. no. MA515739), anti-alpha tubulin antibody clone DM1A (Thermo Fisher cat. no. 62204), Tau monoclonal antibody (T46) (Thermo Fisher cat. no. 136400), anti-Iba-1 (Thermo Fisher cat. no. PA527436), Rab5 (Thermo Fisher, cat. no. MA5-32150), Rab7 (Thermo Fisher, cat. no. PA5-52369), LAMP-2A (Thermo Fisher cat. no. 51-2200), anti-mouse secondary antibody conjugated with Alexa Fluor-488 (Invitrogen, cat. no. A-11001), goat anti-rabbit IgG (H + L) Cross-Adsorbed Secondary Antibody with Alexa Fluor 555 (A-21428), GOXMS ALEXA FLOUR 488 goat anti-rabbit (Thermo Fisher cat. no. A28175), DAPI (Invitrogen), goat anti-ouse secondary antibody peroxidase conjugated (Thermo Fishe 32430), and Prolong Diamond antifade (Thermo Fisher cat. no. P36961).

#### Protein expression and purification

Full-length wild-type Tau protein (hTau40^wt^) was expressed in BL21* cells with 100 µg/ml of ampicillin antibiotic selection and purified with two-step chromatography methods, cation-exchange chromatography, and size-exclusion chromatography [72–74]. Cells were grown at 37°C, scaled up, and harvested after induction with 0.5 mM IPTG for 4 h. Cells were subjected to homogenization to produce cell lysate at 15,000-psi pressure. The cell lysate was subjected to 90°C heating in the presence of 0.5 M NaCl and 5 mM DTT for 20 min to denature structured proteins. The supernatant was collected after centrifugation at 40,000 rpm for 45 min, then put through dialysis overnight in 20 mM MES buffer supplemented with 50 mM NaCl. The supernatant was obtained again after centrifugation at 40,000 rpm for 45 min passed through cation-exchange chromatography. Sepharose fast-flow column was used for chromatography, using 20 mM MES buffer and 50 mM NaCl (buffer A). Elution was done with 20 mM MES buffer and 1 M NaCl (buffer B). Fractions containing Tau proteins were collected after cation-exchange chromatography, which was then concentrated and subjected to size-exclusion chromatography. Size-exclusion chromatography was carried out in the Superdex 75 Hi-load 16/600 column in 1× PBS supplemented with 2 mM DTT. Fractions containing Tau were collected, pooled, concentrated, and the concentration of protein was determined with bicinchoninic acid assay assay.

#### Aggregation assay

Tau protein undergoes aggregation in the presence of poly-anionic reagent such as heparin and arachidonic acid; it is observed by the transition of random coiled structure to the β-sheet formation in protein [75]. In this study, Tau aggregation was induced by heparin (MW-17,500 Da) in the ratio of 1:4 heparin to Tau along with other additives 20 mM BES buffer, 25 mM NaCl, 1 mM DTT, 0.01% NaN_3,_ and PIC. The effect of ALA on Tau aggregation was measured by thioflavin S (ThS) fluorescence assay. ThS is a homogeneous mixture of methylation product of dehydrothiotoluidine in sulfonic acid, which can bind to β-sheet structure. Aggregation kinetics of Tau was studied with 2 µM of Tau and ThS in 1:4 ratios. The excitation wavelength for ThS is 440 nm, and the emission wavelength is 521 nm. Further analysis of data was done using Sigmaplot 10.0.

#### Transmission electron microscopy

Morphological analysis of Tau fibrils and ALA vesicles was studied by (TEM. Also, 2 µM Tau sample was incubated on 400 mesh, carbon-coated copper grid, and stained with 2% uranyl acetate. For ALA vesicles, working concentration of 40 µM was taken for grid preparation [49]. The images were taken with TECNAI T20 120 KV.

#### CD spectroscopy

Conformational changes in Tau from random coiled structure to β-sheet conformation on aggregation of protein were studied using CD spectroscopy. The spectra was collected as previously mentioned in the UV region [76]. The measurement was done in Jasco J-815 spectrometer, and cuvette path length was 1 mm. The measurement was done in the range of 250– 190 nm, with a data pitch of 1.0 nm, and scanning speed was kept 100 nm/min. For measurement, 3 μM sample concentration was taken in phosphate buffer pH 6.8. All the spectra were taken at 25°C.

#### Cell culture and preparation of ALA

N9 (microglia) cells were grown in RPMI media in T25 flask or 60 mm dish supplemented with 10% heat-inactivated serum, 1× penicillin-streptomycin antibiotic solution, and glutamine to maintain the culture. Cells were passaged on 90% confluence using 0.25% trypsin-EDTA solution after washing with PBS. For western blot experiment, cells were seeded in 6-well plates. For ALA preparation, previously published protocol was followed [31]. Briefly, ALA was dissolved in 100% molecular biology grade ethanol and solubilized at 50°C in the stock concentration of 20 mM. The fatty acid solution was prepared freshly before every experiment. According to the previous studies, 40 μM was the working concentration of ALA for carrying further experiments. The final concentration of ethanol in cell culture media was maintained below 0.5% [31,49].

#### Tau internalization

To study the effect of ALA on microglial phagocytosis, N9 cells were treated with extracellular 1 µM monomer and aggregates along with 40 µM ALA. For the immunofluorescence experiment (25,000 cells/well), N9 cells were seeded on 12 mm glass coverslip in 24-well plate. The cells were then incubated with 1 µM Tau monomer and aggregates along with 40 µM ALA for 24 h.. To study the internalization of Tau, N9 cells were treated with similar conditional group. The coverslips were then fixed and stained for immunofluorescence analysis with antibodies Tau T46 (1:400 dilution) and Iba-1 (1:500 dilution). The mounting of coverslips was done with prolonged gold diamond antifade mountant. The intracellular intensity of microglia was calculated from fluorescence images to quantify the internalization, with the help of ZEN 2.3 software. The representation of intracellular intensity was done as a mean intensity of intracellular protein for different groups.

To study the degradation of intracellular Tau after phagocytosis, we have targeted early and late endosomal markers and lysosome marker for final degradation process. The treatment was done as previously mentioned; after 24 h of exposure cells were fixed and stained for immunofluorescence analysis. The analysis of the process of degradation was done by understanding the colocalization of internalized Tau with Rab5 (1:200 dilution), Rab7 (1:200 dilution), and LAMP-2A (1:500 dilution). The intracellular intensities of Rab5, Rab7, and LAMP-2A were studied as a mean intensity of desired protein inside the cell to understand the expression of proteins. The colocalization of internalized Tau was studied with 3-D and orthogonal analysis of immunofluorescence images.

#### Wound-scratch assay

To study the migration of microglia, wound-scratch assay was performed. For the assay (500,000 cells/well), N9 cells were seeded in a 6-well plate and maintained in RPMI media for 24 h till the confluency reached 80%. Scratch was created with sterile 200 μl pipette tip, followed by treatment with groups as mentioned previously. Cells were incubated further for 24 h to study the migration of N9 cells into the wound. A number of cells that migrated into the wound were calculated for five different areas of culture, and the average was calculated to quantify the migration. The graph is plotted as the number of cells traveling into the wound at different time intervals of 0, 6, 12, and 24 h [77].

#### MTOC reorientation analysis

To study the polarization of MTOC around the nucleus, immunofluorescence experiment has been performed with N9 cells after the desired treatment of Tau monomer, aggregates, and ALA. After 24 h, the cells were fixed for immunofluorescence staining. The MTOC positions were analyzed by α-tubulin (1:200) staining. The anterior, posterior, and lateral positions of MTOC were counted with respect to the nucleus, which is denoted by DAPI stain. The percentage of MTOC positions was calculated in 10 different fields containing 5–7 cells each.

#### Immunofluorescence analysis

N9 cells were passaged in RPMI media supplemented with 10% FBS and 1× penicillin-streptomycin. For immunofluorescence studies, 25,000 cells were seeded on 12 mm coverslip (Bluestar) in 24-well plate. Supplemented with 0.5% serum-deprived RPMI media for the desired treatment. The treatment was given for 24 h. Cells were then fixed with chilled absolute distilled methanol for 20 min at −20°C, then washed with 1× PBS thrice. Permeabilization was carried out using 0.2% Triton X-100 for 15 min, washed three times with 1× PBS, followed by blocking with 2% serum in 1× PBS for 1 h at room temperature. Primary antibody treatment was given to cells overnight at 4°C in 2% serum in 1× PBS in a moist chamber. The next day, cells were washed with 1× PBS thrice. Then these were incubated in the desired secondary antibody in 2% serum at 37°C for 1 h. Further cells were washed with 1× PBS three times and counterstained with DAPI (300 nM). Mounting of coverslip was done in prolonged gold diamond antifade mountant. Images were observed under a 63× oil immersion lens in Axio observer 7.0 Apotome 2.0 Zeiss microscope.

#### Western blot

For detection of protein levels in cells (300,000 cells/well), N9 cells were seeded in 6-well plate. Treatment exposure was followed by washing with 1× PBS. Cell lysis was carried out using radioimmunoprecipitation (RIPA) assay buffer containing protease inhibitors for 20 min at 4°C. The cell lysate was centrifuged at 12,000 rpm for 20 min. Protein concentration was checked by Bradford’s assay, and equal amount of 75 µg total proteins for all the treatment groups was loaded on polyacrylamide gel electrophoresis of range 4–20% and the gel was electrophoretically transferred to polyvinylidene difluoride membrane and kept for primary antibody Rab5, Rab7, and LAMP-2A (1:1000 dilution) binding overnight at 4°C. After the incubation, washing of blot was carried out three times with 1× PBST (0.1% Tween-20). The secondary antibody was incubated for 1 h at room temperature. Then the membrane was developed using chemiluminiscence detection system. The relative quantification of protein was carried out with loading control -β-actin (1:5000) in each treatment group.

#### Statistical analysis

All the experiments were performed three times. The data was analyzed using SigmaPlot 10.0, and the statistical analysis was carried out by Student’s *t*-test (*t*-test: two samples assuming equal variance) to calculate the significance between the conditions. The *P* values for each condition are mentioned on the respective bar graph. The significance of data in the the form of star values is as follows: ns, non-significant; * *P* ≤ 0.05; ** *P* ≤ 0.01; *** *P* ≤ 0.001. All the graphs are plotted as mean+standard error and the data showing *P* value ≤0.05 considered as significant. The quantification of levels of intracellular proteins in immunofluorescence experiments was carried out by measuring the mean intensity of the desired protein and the corresponding area of microglia with Zeiss ZEN 2.3 software for image processing. The calculation of intracellular intensity was done with the help of contour tool from the software. The intensity analysis of protein bands from western blot experiment was done with the help of ImageJ software. The band intensity was compared with loading control protein (β-actin) to understand the expression pattern. The graph is plotted as a normalized intensity of protein with loading control.

## Supplementary Material

Supplemental Material
