# Supplementary material for: α– Linolenic acid modulates phagocytosis and endosomal pathways of extracellular Tau in microglia
Source: Cell Adh Migr. 2021 Mar 16;15(1):84–100. doi: 10.1080/19336918.2021.1898727 (PMC7971307; doi:10.1080/19336918.2021.1898727)
Supplement: Supplemental Material [file KCAM_A_1898727_SM1104.docx]

**Supplementary information**

**α-Linolenic acid modulates phagocytosis and endosomal pathways of extracellular Tau in microglia**

**Smita Eknath Desale^1, 2^ and Subashchandrabose Chinnathambi^1, 2, *^**

^1^Neurobiology Group, Division of Biochemical Sciences, CSIR-National Chemical Laboratory,

Dr. Homi Bhabha Road, Pune, 411008, India

^2^Academy of Scientific and Innovative Research (AcSIR), Ghaziabad, 201002, India

^*^To whom correspondence should be addressed**: Prof. Subashchandrabose Chinnathambi**, Neurobiology group, Division of Biochemical Sciences, CSIR-National Chemical Laboratory (CSIR-NCL), Dr. Homi Bhabha Road, 411008 Pune, India, Telephone: +91-20-25902232, Fax. +91-20-25902648. Email: [**s.chinnathambi@ncl.res.in**](mailto:s.chinnathambi@ncl.res.in)

**Author Details**

Smita Eknath Desale, email: [se.desale@ncl.res.in](mailto:se.desale@ncl.res.in)

Subashchandrabose Chinnathambi, email: [s.chinnathambi@ncl.res.in](mailto:s.chinnathambi@ncl.res.in)

**Supplementary figure 1**. **Internalization of extracellular Tau in microglia on ALA exposure.** The N9 cells exposed with ALA and Tau species are analyzed for internalization of Tau via fluorescence microscopy Tau (red), Iba-1 (green), DAPI (blue). The scale bar is 20 µm.

**Supplementary figure 2**. **Repolarization axis of Microtubule organizing center (MTOC) in microglia on ALA exposure.** The repolarization of MTOC was studied with the help of fluorescence microscopy α-tubulin (red), DAPI (blue). The scale bar is 20 µm.
